# Determination of heavy metals (Pb, Cr, As, Hg, and Cd) into the body organs of selected fish, water, sediment, and soil samples from Head Punjnad and Head Taunsa, Punjab, Pakistan

**DOI:** 10.1371/journal.pone.0288163

**Published:** 2023-09-05

**Authors:** Khalid Javed Iqbal, Noor Khan, Mahroze Fatima, Mushtaq Hussain Lashari, Muhammad Asad, Asma Ashraf, Huma Fatima, Hamid Majeed, Irfan Baboo, Khalid Jamshed Iqbal, Muhammad Asghar, Usama Saeed, Wazir Ali, Sadia Nazir, Sheeza Bano, Ayesha Tanveer

**Affiliations:** 1 Department of Zoology, The Islamia University of Bahawalpur, Punjab, Pakistan; 2 Institute of Zoology, University of the Punjab, Lahore, Pakistan; 3 Department of Fisheries and Aquaculture, University of Veterinary and Animal Sciences, Lahore, Pakistan; 4 Department of Zoology, Division of Science and Technology, University of Education, Lahore, Pakistan; 5 Department of Zoology, Women University Mardan, Mardan, Pakistan; 6 Department of Food Science and Technology, Cholistan University of Veterinary and Animal Sciences Bahawalpur, Bahawalpur, Pakistan; 7 Department of Zoology, Cholistan University of Veterinary and Animal Sciences Bahawalpur, Bahawalpur, Pakistan; 8 Department of Chemistry, Khawaja Fareed University of Engineering and Information Technology, Rahim Yar Khan, Pakistan; 9 Department of Biology, University of Copenhagen, København, Denmark; Sher-e-Kashmir University of Agricultural Sciences and Technology of Kashmir, INDIA

## Abstract

The present study was conducted on Head Punjnad (HP) and Head Taunsa (HT) to evaluate the contamination of Pb, Cr, As, Hg, and Cd in water, soil, sediment, fish as a whole and fish organs. Fish, water, soil and sediment samples were collected from different sites of HT and HP on a monthly basis for 8 months. Heavy metals in water, soil, and sediment were determined by a polarized Zeeman atomic absorption spectrophotometer and in fish and fish organs by an atomic absorption spectrophotometer. Contamination of Cd, Hg, and As was significantly (P<0.05) higher in water of HP as compared to HT, while Cr showed a non-significant (P>0.05) difference at HP and HT. Pb was significantly (P<0.05) higher in water of HT as compared to HP. In the case of soil, Cd, Hg, and Pb were higher at HT as compared to HP, while As and Cr were significantly (P<0.05) higher at HP as compared to HT. In sediment, contamination of Cd, Hg, and As were significantly (P<0.05) higher at HP as compared to HT, while the Cr difference was non-significant (P>0.05) but Pb showed a significantly (P<0.05) higher value at HT than HP. Cd accumulation in different fish species was recorded as *R*. *rita ˃O*. *niloticus ˃C*. *marulius ˃S*. *sarwari ˃C*. *idella ˃C*. *catla ˃N*. *notopterus ˃E*. *vacha ˃L*. *rohita ˃C*. *carpio*, respectively. Hg as *O*. *niloticus* ˃*S*. *sarwari* ˃*R*. *rita* ˃*C*. *marulius* ˃*C*. *catla* ˃*N*. *notopterus* ˃*E*. *vacha* ˃*L*. *rohita* ˃*C*. *carpio* ˃*C*. *idella*, respectively. As as *O*. *niloticus* ˃*R*. *rita* ˃*S*. *sarwari* ˃*C*. *marulius* ˃*C*. *catla* ˃*C*. *carpio* ˃*N*. *notopterus* ˃*C*. *idella* ˃*E*. *vacha* ˃*L*. *rohita*, respectively. Cr accumulation recorded as *L*. *rohita* ˃*C*. *idella* ˃*O*. *niloticus* ˃*C*. *marulius* ˃*E*. *vacha* ˃*R*. *rita* ˃*C*. *catla* ˃*C*. *carpio* ˃*S*. *sarwari* ˃*N*. *notopterus*, respectively. Pb accumulation in different fish species was recorded as *C*. *idella* ˃*C*. *carpio* ˃*N*. *notopterus* ˃*L*. *rohita* ˃*O*. *niloticus* ˃*C*. *marulius* ˃*R*. *rita* ˃*S*. *sarwari* ˃*E*. *vacha* ˃*C*. *catla*, respectively. Cd accumulation in different organs was recorded as kidney ˃liver ˃gills ˃muscle ˃skin ˃scale. Hg accumulation in different organs was recorded as kidney ˃gills ˃liver ˃skin ˃muscle ˃scale. As accumulation in different organs was recorded as kidney ˃liver ˃gills ˃muscle ˃skin ˃scale. Cr accumulation in different organs was recorded as gills ˃ liver ˃skin ˃muscle ˃kidney ˃scale. Pb accumulation in different organs was recorded as gills˃ kidney˃ skin˃ liver˃ muscle˃ scale.

## Introduction

Metals with an atomic number of more than 20 and possessing an elemental density of more than 5gcm^-1^ that are naturally occurring are called heavy metals. Different terminologies are used for heavy metals like trace metals, hazardous or toxic metals, micronutrients, non-essential and essential metals, and trace elements [1]. These heavy metals are commonly found in water and are required by aquatic organisms in small quantities for their normal biological and physiological functions. However, the diverse effects of heavy metals make them highly detrimental substances, particularly when present in high concentrations, as they can adversely impact the blood and organs of fish [2]. As human civilization progresses, our actions have been altering the natural aquatic ecosystem in various ways. These changes include the accumulation of pollutants, environmental degradation, and shifts in natural patterns [3–5]. Examples of such pollutants include industrial discharges [6], heavy metals [7], pharmaceuticals, and agricultural pesticides [8, 9], all of which contribute to the degradation of the ecosystem.

The industrial revolution in Pakistan is leading to a rapid increase in aquatic pollution across various regions. One significant contributor to this pollution is the presence of heavy metals, which have a long-lasting impact on the aquatic ecosystem. These metals are released from various sources and exist in combined forms in the aquatic environment. When present in combination, metals exhibit diverse effects compared to their individual states. To assess the health of a specific body of water, fish can serve as reliable indicators [10]. Pakistan, like many developing countries, faces numerous environmental challenges, including deteriorating water quality. Limited resources and a lack of technology hinder the proper disposal of waste generated from human activities [1]. Consequently, simple remediation techniques are ineffective in removing these heavy metals. Multiple pathways serve as entry points for these metals into the human body, such as ingestion, skin contact, and inhalation. Agricultural runoff, urban discharge, and wastewater release into water bodies contribute to the contamination of both sediments and water [11, 12].

Metals are categorized into two groups: essential and non-essential, based on their importance for the growth and development of plants, animals, and humans. Certain metals, including nickel (Ni), chromium (Cr), cadmium (Cd), and lead (Pb), are categorized as non-essential metals, while copper (Cu), manganese (Mn), iron (Fe), and zinc (Zn) are considered essential metals that should be present within acceptable limits [1]. Among these metals, mercury (Hg) is particularly destructive and is commonly found in urban and industrial wastewater. Its primary sources include mining, dental preparation, pulp, cement, and paper industries, contributing to environmental contamination [13]. Hexavalent chromium is highly toxic, especially in its oxidized form, causing harm to embryos and even leading to cancer in aquatic organisms [2]. Heavy metals have attracted significant attention in aquatic research as they are often identified as major toxic agents affecting aquatic organisms, particularly fish [5, 14]. For the propagation and survival of aquatic organisms and fisheries, good water quality is an essential factor [15–18]. Aquatic pollution is well-known for contaminating sediments and impacting various aquatic life forms such as fish, aquatic plants, shellfish, and overall water quality [19–21]. The bioaccumulation of heavy metals in fish chiefly depends on the type of heavy metal, fish species, and fish tissues. As compared to sediments, microflora, and water, fish in aquatic environments accumulate large proportions of heavy metals in their tissues. The rate of accumulation of heavy metals in the tissues of fish increases with the number of heavy metals in water [22]. It is crucial to understand and address the presence of heavy metals in aquatic environments due to their potential harm to organisms and ecosystems. The purpose of this study is to recognize the sources and effects of these pollutants. So, we can work towards mitigating their impacts and preserving the health and balance of aquatic ecosystems.

## Materials and methods

### Study area

**Site 1:** The Head Punjnad (HP) is also known as Punjnad River and descends name that literally means ‘Fiver Rivers’ implying thereby the confluence point of five great rivers of Punjab (land of five rivers). This is the termination point of upper Indus basin and the beginning of lower Indus basin. The Punjnad Headwork (also known as Punjnad Barrage) is the confluence point of three local canals: 1) Punjnad canal; 2) Abbassia canal and 3) Abbassia link canal. These canals are the main source of irrigation for mega districts of Bahawalpur and Rahim Yar Khan as well as northern part of Sindh province implying thereby greater importance of useable water quality. The location coordinates are 29°20’47.3"N and 71°1’11.55"E. This area is generally characterized by large-scale industrial and agricultural activities that are the major source of pollutants drained into the Head Punjnad.

**Site 2:** Head Taunsa (Barrage) is another sampling site which is located at Indus River in Muzafargarh, Punjab. It was designated a Ramsar site on March 22, 1996. Further it serves 2.351 million acres besides diverting flows from Indus River to the Chenab river through Taunsa -Punjnad (TP) Link Canal. The location coordinates are 30°30’49.81"N and 70°51’23.75"E.

### Sample collection

Ten fish species viz. Thaila (*Catla catla*), Rohu (*Labeo rohita*), Grass carp (*Ctenopharyngodon idella)*, Common carp (*Cyprinus carpio*), Nile tilapia (*Oreochromis niloticus)*, Jhali (*Eutropiichthys vacha)*, Khagga (*Rita rita)*, Great snakehead (*Channa marulius)*, Singhari (*Sperata sarwari*) and Feather Back (*Notopterus notopterus)* were collected during August-December 2017 (5 months sampling) from HP and HT by using a cast net with help of local fisherman and identified on the basis of their morphology [23]. In order to ensure the well-being of the fish, they were anesthetized using MS-222 until they reached a surgical level of anesthesia, resulting in a loss of response to noxious stimuli. All protocol and procedure was approved by guidelines for care and use of laboratory animals committee of The Islamia University Bahawalpur, Pakistan (No. Dr/793) which aim to minimize pain and distress. This allowed for the collection of fish organs with minimal pain and distress. Throughout the sampling and experimental procedures, efforts were made to alleviate suffering by taking great care to minimize handling stress by using appropriate nets and tools, reducing the risk of injury and stress associated with capture and handling. Each month, six fish samples of each fish species as well as equal number of soil, sediment and water samples were collected. Fish samples were put in sterile polythene bags, while, water samples were collected in pre-rinsed plastic bottles with 0.01N nitric acid and with the help of plastic spoon soil and sediment samples were collected from those points where the water samples were taken and all samples (fish, soil, sediment, and water) were transported with ice box on the same day to the laboratory. Sediment and water samples preserved at 4°C and fish samples at -4°C for further laboratory analyses, which was performed by using the Zeeman Atomic Absorption Spectrometer (Z-5000, Hitachi Japan).

### Sample preparation

In order to detect metal presence and their level in fish, soil, sediment, and water samples were processed for metal detection in spectrophotometer. Firstly, the fishes were degutted with sterile scissors to remove target organs (gills, kidney, liver, muscle, scale and skin) and obtained samples were preserved in iceboxes at -18°C for further process. The target organs were properly dried in an oven at 65°C for 24 hours followed by burning in a furnace at 700°C to 1000°C for 90 minutes. Later, ash contents were mixed in concentrated solutions of sulphuric acid (H_2_SO_4_) and nitric acid (HNO_3_). Finally, this solution was heated for two hours and then mixed with distilled water and filtered prior to heavy metal analysis using atomic absorption spectrophotometer [24, 25]. Briefly, for a water sample, 450 ml water sample along with 6 ml nitric acid was added in a flask and heated at 100 °C. Later hydrogen peroxide (H_2_O_2_) was added and filtered prior to analysis using atomic absorption spectrophotometer [26]. Soil and sediment samples were kept in a flask in nitric acid and hydrogen peroxide was added dropwise. Samples were kept at room temperature and filtered later. Distilled water was added and then stored at room temperature for the determination of metals [27].

### Statistical analyses

Independent sample T-test was used to compare heavy metal concentrations in water of head Punjnad and head Taunsa. One-way ANOVA was used to compare the means of heavy metals in different fish species, water and sediment samples. For statistical inferences, we used SAS (v. 9.1) and PAST software.

## Results and discussions

### Heavy metals concentration in water

In 2 selected sites, five heavy metals were detected in water (Table 1). The results showed highest Cd and Pb concentrations in HP (26.06, 2.22ppb) and lowest in HT (12.71, 2.41ppb) in water. These results were in line with [27], who reported heavy metal concentration in Kali River (Muzaffarnagar district) ranged from 0.001–0.024ppm and 0.001–0.34ppm (1–24 and 1–340ppb). These showed an average concentration of Cd and Pb set by WHO and US-EPA, which is low compared to Kurram river 0.04–0.14 and 2.50–9.50ppm (40–140 and 2500–9500ppb) [1]. The study reveals a meager amount of Cr (.72ppb) and As (9.35ppb) metal in water at all sites (Table 1). In contrast, the study on Kali river reported Cr concentration as 0.002–0.087ppm (2–87ppb) [28], Kurram river 2.50–8.00ppm (2500–8000ppb) [1], industrially polluted water of river Ravi 0.39–0.50ppm (390–500ppb) near Lahore [22]. Moreover, As concentration as 0.01ppm (10ppb) in the Taunsa barrage [15]. Hg concentration in water was (3.06, 1.09ppb) in HP and HT while in opposite [29] reported the highest concentration of Hg 0.050–0.056ppm (50–56ppb) in river Niger. Similarly, [30] also documented highest concentrations of Cr and Pb (2.5, 1.02 mg/L) in Dhaleshwari river in Bangladesh. The sites that show high metal concentrations are linked with the discharge of waste material and agriculture runoff. Also, leachates and dumping sites contribute to the metal contamination of sites. Another reason is that people who live closer to these sites discharge waste water and home solid waste. Furthermore, the use of fertilizers closer to these sites also causes water contamination. The sites that are far away from the source point showed low metal contamination concentrations, such as chromium. Nowadays, pesticide and fertilizers use for plant growth, and disease control is common in modern agriculture, which contributes to constant freshwater body degradation [31].

**Table 1:** Comparative study of heavy metal accumulation in water at HP and HT.

Metals	Head Punjnad	Head Taunsa
	Water	Water
**Cd (ppb)**	26.06±4.08^b^	12.71±.43^a^
**Hg (ppb)**	3.06±.16^d^	1.09±.01^a^
**As (ppb)**	9.35±1.09^d^	6.66±.21^c^
**Cr (ppb)**	0.72±.06^a^	0.65±.02^a^
**Pb (ppb)**	2.22±.18^b^	2.41±.08^c^

Means values with a different superscript in a row showed a significant (P<0.05) difference

### Heavy metals concentration in soil and sediments

Sediments act like a sink and are also a source of water contamination with heavy metals. To assess the organic or inorganic pollution load in water, sediments play a critical role [32]. [33] determined heavy metal concentrations in water, sediments, and aquatic vegetation at Site Burley Griffen; they found sediments contained a higher concentration of heavy metal than water or aquatic plants. Our study also confirmed higher heavy metal concentrations in sediments (Table 2) than in water (Table 1). As concentrations were 3.34 and 2.02 ppb at HP and HT in soil. The highest concentrations of Cd were in HT (67.64 ppb) and the lowest in HP (40.26 ppb), while Cr and Pb concentrations in HP and HT (1.47, 1.25 ppb and 1.80, 1.92 ppb) were at both sites in soil., which are lower compared to the findings of [1], who reported Cd, Cr, and Pb metal ranges (0.10–0.15 ppm), (10.25–18.5 ppm) and (1.00–12.5 ppm) at Kurram river [28]. Detected Cd, Cr, and Pb ranged from 0.11–3.38 ppm (110–3380 ppb), 0.35–20.11 ppm (350–20110 ppb) and 14.22–81.53 ppm (14220–81530 ppb) from different sites of the Kali river. Also, [30] documented highest concentrations of Cr and Pb (96.02 and 23.69 mg/kg in monsoon) in Dhaleshwari river in Bangladesh. Hg concentration in soil was (1.96, 2.37 ppb) in HP and HT, while in opposite [29], reported the highest concentration of Hg was 0.560–0.566 ppm (560–566 ppb) in river Niger.

**Table 2:** Comparative study of heavy metals accumulation in soil and sediment at different study sites.

Metals	Head Punjnad		Head Taunsa	
	Soil	Sediment	Soil	Sediment
**Cd (ppb)**	40.26±1.02^c^	97.13±25.61^a^	67.64±.71^d^	65.12±23.46^b^
**Hg (ppb)**	1.96±.07^b^	2.64±1.63^a^	2.37±.04^c^	1.69±1.10^b^
**As(ppb)**	3.34±.11^b^	2.11±1.14^a^	2.02±.09^a^	0.92±0.63^b^
**Cr (ppb)**	1.47±.04^c^	590.00±88.84^a^	1.25±.05^b^	561.39±278.59^a^
**Pb (ppb)**	1.80±.01^a^	1091.41±647.78^b^	1.92±.01^a^	1497.02±282.00^a^

Means values with a different superscript in a row showed a significant (P<0.05) difference

### Heavy metal accumulation in different fish species

In our study, we found variations in the concentrations of heavy metals among different fish species in various rivers. The highest concentration of cadmium (Cd) was recorded in *R*. *rita* (112.02ppb), while *C*. *carpio* had the lowest concentration (43.26ppb) (Table 3). In the upper Indus river, *C*. *mrigala* showed a high accumulation of Cd (0.97ppm or 970ppb), whereas *L*. *calbasu* had a lower concentration (0.14ppm or 140ppb). In the lower Indus river, *C*. *mrigala* contained a high amount of Cd (0.89ppm or 890ppb), whereas *C*. *chitala* had a low amount (0.03ppm or 30ppb). In the Chenab river, *C*. *carpio* exhibited a high Cd level (1.48ppm or 1480ppb), while *C*. *garua* had a low level (0.22ppm or 220ppb). Similarly, in the Kabul river, *C*. *carpio* showed high contamination of Cd (1.13ppm or 1130ppb), while *T*. *macrolips* had a lower level (0.08ppm or 80ppb) [34].

**Table 3:** Comparative study of heavy metals accumulation in different fish species.

Species	Cd (ppb)	Hg (ppb)	As (ppb)	Cr (ppb)	Pb (ppb)
***C*. *idella***	87.42±20.66^bcd^	0.80±0.64^d^	1.00±0.14^cde^	653.33±73.12^a^	1795.00±454.79^a^
***C*. *carpio***	43.26±19.48^f^	1.03±0.48^d^	1.57±0.60^bc^	581.40±269.86^a^	1528.40±404.79^ab^
***E*. *vacha***	68.50±7.26 ^de^	1.26±1.018^d^	0.55±0.11^de^	598.33±48.34^a^	910.00±619.26^cd^
***R*. *rita***	112.02±20.74^a^	2.81±2.02^b^	3.20±0.47^a^	591.00±101.37^a^	1179.00±515.50^bcd^
***N*. *notopterus***	74.09±17.70^cde^	1.65±0.37^cd^	1.31±0.37^bcd^	316.00±299.82^b^	1486.00±330.16^ab^
***L*. *rohita***	64.66±22.18^e^	1.23±0.76^d^	0.43±0.26^e^	671.25±73.65^a^	1444.94±388.83^ab^
***S*. *sarwari***	89.89±16.14^bc^	3.27±1.40^ab^	2.07±1.56^b^	528.89±244.32^a^	1032.22±637.29^bcd^
***C*. *marulius***	104.83±27.49^ab^	2.67±0.55^bc^	1.68±0.38^bc^	613.13±130.01^a^	1185.63±507.48^bcd^
***C*. *catla***	74.68±20.39^cde^	2.37±1.45^bc^	1.59±0.59^bc^	583.75±34.62^a^	705.00±630.42^d^
***O*. *niloticus***	109.43±21.27^a^	3.95±0.57^a^	3.96±0.52^b^	645.00±97.78^a^	1424.00±552.81^abc^

Mean values with a different superscript in the column showed a significant (P<0.05) difference

Regarding mercury (Hg) concentrations, *O*. *niloticus* recorded the highest concentration (3.95ppb), while *C*. *idella* had the lowest (0.80ppb). As for arsenic (As), *R*. *rita* had the highest concentration (3.20ppb), while *L*. *rohita* had the lowest (0.43ppb) (Table 3). In a previous study [34] conducted on the main rivers of Pakistan, *L*. *rohita* showed a high accumulation of As (0.67ppm or 670ppb) in the upper Indus river, whereas *C*. *mrigala* had a lower concentration (0.08ppm or 80ppb). In the lower Indus river, *L*. *rohita* contained a high amount of As (2.50ppm or 2500ppb), while *S*. *seenghala* had a low amount (0.13ppm or 130ppb). In the Chenab river, *C*. *carpio* exhibited a high As level (2.98ppm or 2980ppb), whereas *C*. *reba* had a low level (0.46ppm or 460ppb). Similarly, in the Kabul river, *C*. *carpio* showed high contamination of As (0.86ppm or 860ppb), while *G*. *stocki* had a lower level (0.008ppm).

The concentration of chromium (Cr) varied among species as well. *L*. *rohita* had the highest concentration (671.25ppb), while *N*. *notopterus* had the lowest (316ppb) (Table 3). In the upper Indus river, *L*. *calbasu* showed a high accumulation of Cr (0.17ppm or 170ppb), whereas *R*. *rita* had a lower concentration (0.03ppm 30ppb). In the lower Indus river, *L*. *rohita* contained a high amount of Cr (0.56ppm or 560ppb), while *S*. *seenghala* had a low amount (0.002ppm or 2ppb). In the Chenab river, *M*. *aramyus* exhibited a high Cr level (0.64ppm or 640ppb), while *C*. *garua* had a low level (0.28ppm or 280ppb). Similarly, in the Kabul river, *C*. *carpio* showed high contamination of Cr (0.96ppm or 960ppb), whereas *G*. *stocki* had a lower level (0.03ppm or 30ppb) [34].

Lead (Pb) concentrations also varied among fish species. The concentration of lead (Pb) was found to be high in *C*. *idella* (1795ppb) and low in *C*. *catla* (705ppb) (Table 3). Another study [34] conducted on three rivers in Pakistan reported high accumulation of Pb in *C*. *mrigala* (1.92ppm or 1920ppb) and low accumulation in *R*. *rita* (0.31ppm or 310ppb) in the upper Indus River. In the lower Indus river, *C*. *mrigala* contained a high amount of Pb (4.89ppm or 4890ppb), while *S*. *seenghala* had a low amount (0.29ppm or 290ppb). In the Chenab river, *M*. *aramyus* exhibited a high Pb level (5.93ppm or 5930ppb), while *C*. *reba* had a low level (0.18ppm or 180ppb). Similarly, in the Kabul river, *C*. *carpio* showed high contamination of Pb (2.34ppm or 2340ppb), whereas *A*. *seenghala* had a lower level (0.01ppm or 10ppb) [34].

### Heavy metal concentration in different fish organs

The concentrations of Cd, Hg, and As (100.25, 3.75, and 2.23 ppb) were recorded highest in the kidney and lowest (26.20, 0.26, and 0.65 ppb) in the scales of different fishes. While Cr and Pb concentrations (649.58 and 1370 ppb) were recorded highest in the gills and lowest (430 and 1076.88 ppb) in the scales (Table 4) [35]. Conducted a study on heavy metals in organs of *L*. *rohita* and *W*. *attu* from the Indus river, Pakistan, in which he documented the contamination of Pb, As, and Cr in gills (3.46, 0.61, and 3.26 μg/g), muscles (1.56, 0.46, and 0.67 μg/g), liver (2.45, 0.95, and 2.14 μg/g) and skin (2.65, 0.96, and 1.45 μg/g) in *L*. *rohita*. Pb was highest in gills (3.46 μg/g) and lowest in muscles (1.56 μg/g). As was highest in skin (0.96 μg/g) and lowest in muscle (0.46 μg/g). Cr was highest in gills (3.26 μg/g) and lowest in muscles (0.67 μg/g) of *L*. *rohita*. In *W*. *attu*, Pb (3.64 μg/g) was highest in gills while lowest in muscles (1.95 μg/g). As (0.88 μg/g) was highest in skin while lowest in muscle (0.27 μg/g). Cr was highest in skin (1.43 μg/g) and lowest in muscle (0.55 μg/g) [13]. Reported the bioaccumulation of Hg in different organs of *L*. *rohita* and tilapia in summer and winter from the river Ravi and urban sewage of Lahore. Hg in liver, kidney, and muscle (0.31, 0.38, and 0.24 ng/g) in summer was highest as compared to winter (0.29, 0.33, and 0.21 ng/g) but in gills, it was the opposite: highest in winter (0.31 ng/g) and lowest in summer (0.23 ng/g). In tilapia, Hg concentrations were highest in liver, kidney, gills, and muscle (0.26, 0.33, 0.28, and 0.22 ng/g) as compared to winter (0.22, 0.11, 0.24 and 0.20 ng/g) [2]. Documented Pb and Cr contamination in different organs of *C*. *carpio* and *L*. *rohita* from Sardaryab, Khyber Pakhtunkhwa. In gills, liver, and muscles of *C*. *carpio* Pb was (0.014, 0.142, and 0 μg/g) and Cr was (0.154, 0.188, and 0.024 μg/g). In *L*. *rohita*, gills, liver, and muscles contain Pb (0.024, 0.161, and 0 μg/g) and Cr (0.133, 0.165, and 0.019 μg/g).

**Table 4:** Comparative study of heavy metals accumulation in different fish organs.

Organs	Cd (ppb)	Hg (ppb)	As (ppb)	Cr (ppb)	Pb (ppb)
**Gills**	95.25±21.84^ab^	2.60±1.01^b^	1.63±0.95^ab^	649.58±91.25^a^	1370.00±14.14^a^
**Kidney**	100.25±25.78^a^	3.75±1.11^a^	2.23±1.54^a^	462.25±47.31^ab^	1362.10±629.11^a^
**Liver**	98.59±27.50^a^	3.13±1.10^ab^	1.88±1.07^ab^	613.22±64.01^ab^	1265.22±62.52^a^
**Muscle**	80.83±22.86^b^	1.40±1.17^c^	1.38±0.93^bc^	545.77±42.68^ab^	1226.54±54.09^a^
**Scale**	26.20±8.38^d^	0.26±0.08^d^	0.65±0.43^c^	430.00±48.83^b^	1076.88±91.09^a^
**Skin**	62.02±17.47^c^	1.43±1.33^c^	1.26±1.01^bc^	604.58±48.14^ab^	1307.50±80.42^a^

Means values with a different superscript in the column showed a significant (P<0.05) difference

## Conclusion

In conclusion, this study reported the levels of five heavy metals (Cd, Hg, As, Cr, and Pb) in water and sediment samples collected from two famous tourist spots i.e., Head Punjnad and Head Taunsa. We also investigated loads of the heavy metals in ten different fish species considered important as commercial species and preferred by the local and visiting people. We also targeted six different fish organs to estimate the heavy metals’ presence and concentrations, and the organs included gills, kidney, liver, muscle, scale, and skin. Our outcomes indicated that Cd was the most prevalent metal in both sites, with head Taunsa as the most polluted site. Cr concentration was found to be the lowest at both study sites. In the case of metals concentration in fish, Pb was the highest, followed by Cr and Cd. The *C*. *idella* accumulated the highest levels of Pb, while *L*. *rohita* absorbed Cr the most. Cd, Hg, and As were observed as the highest in *O*. *niloticus*. Fish gills and kidneys displayed the highest loads of Pb and Cr followed by Cd. Overall, our findings exhibited that head Taunsa is more polluted than head Punjnad. Simultaneously, almost all the commercially important fish species are at a higher risk of high bioaccumulation of heavy metals that may cause severe human health crises if the fish consumption from the polluted sites remains continued. This study achieved all its targeted investigations. The results may help revise the controlling strategies of the industrial effluents so that we may save our freshwater fish species and allied industries.
